# Living Mulch and Organic Fertilization to Improve Weed Management, Yield and Quality of Broccoli Raab in Organic Farming

**DOI:** 10.3390/plants9020177

**Published:** 2020-02-01

**Authors:** Mariano Fracchiolla, Massimiliano Renna, Massimiliano D’Imperio, Cesare Lasorella, Pietro Santamaria, Eugenio Cazzato

**Affiliations:** 1 Department of Agricultural and Environmental Science, University of Bari “Aldo Moro”, 70126 Bari, Italy; mariano.fracchiolla@uniba.it (M.F.); cesare.lasorella@uniba.it (C.L.); pietro.santamaria@uniba.it (P.S.); 2 Institute of Sciences of Food Production, CNR, National Research Council of Italy, 70126 Bari, Italy; massimiliano.dimperio@ispa.cnr.it

**Keywords:** *Brassica rapa* L. subsp. *sylvestris* L. Janch. var. *esculenta* Hort, colour, cover crop, nitrate, sustainability, *Trifolium subterraneum*, *Trifolium repens*

## Abstract

Living mulch gives many benefits to agro-ecosystems such as erosion control, nitrogen fixation and nutrient recycling, increasing of organic matter, weed and pest control, and increasing of soil organism. The experiment, carried out in Puglia, Southern Italy on transplanted broccoli raab (cv. Grossa fasanese), evaluated four soil management systems (SMSs): *Trifolium subterraneum* and *T. repens* used as living mulch, undisturbed weedy, and conventional tillage. For each SMS, four rates of nitrogen and phosphorous (NP0, NP1, NP2, and NP3) were supplied, using an organic fertilizer. The following data were collected: weed infestation, leaf chlorophyll in the plants (as SPAD units), weight, diameter, and colour of the inflorescences, anion and Mg, Fe, Na, K, Ca content. Fertilization showed prominent effects on most of parameters evaluated. The Sufficient Index of broccoli raab plants was higher in fertilized plots. With the increasing of fertilization rates, weight of primary inflorescences and the marketable yield linearly increased, confirming the great influence of nitrogen fertilization on the yield of *Brassicaceae* vegetables and highlighting the importance of combining living mulch and fertilization. By increasing fertilization rates, some elements, such as Mg and Fe, increased, whereas a decrease of Na, K, and Ca was observed. The nitrate content in the inflorescences was different only between the fertilized and unfertilized plots, although it was very low. In NP2 and NP3 a greener colour was found. Living mulch did not clearly affect quality and yield of broccoli raab but was effective in weed control. Results show the positive effects of living mulch and organic fertilization in the sustainable production of broccoli raab.

## 1. Introduction

One of the main objectives of sustainable farming is the safeguard of soil health in agro-ecosystems. This principle is mentioned in Regulation 2018/848 of the European Parliament and of the Council [1], containing the basic objectives and general principles of organic farming, as well as in the “Soil Thematic Strategy”, which sets out common principles for protecting soil across the European Community [2]. Soil health can be defined as “*The ability of a specific type of soil to function within natural or managed ecosystem boundaries, to sustain plant and animal productivity, maintain or improve air quality and water to support human health and liveable*” [3].

Appropriate soil management and tillage systems are able to take care of the soil health, plant growth, and environment simultaneously [4]. In this framework, cover cropping can be an important component of sustainable agriculture, because of its positive impact on various physical and biological processes of soil. Thus, there is a growing interest in considering cover crops as a component of alternative practices to conventional agriculture [5]. 

The main benefits of cover crops in agro-ecosystems are erosion control, nitrogen fixation and nutrient recycling, increasing of organic matter, and weed and pest control [5,6,7,8], as well as the increase of soil organism [9]. Cover crops are grown between or during primary cropping seasons; in this last case, they act as living mulch, which is a companion crop planted at the same time of the main crop and allowed to grow during the growing season as well as after the crop is harvested [10].

There is much interest in living mulch systems in several crops. For example, Adamczewska-Sowińska et al. [11] reported that white clover increased the yield of tomato, although it needed to be periodically mowed in order to avoid competition with the crop. In addition, Wojciechowski et al. [12] reported positive effects of living mulch with white clover and perennial ryegrass on soil structure in terms of stability of soil aggregates. White clover has been reported to increase arbuscular mycorrhiza and yield of corn, because its living mulch acts as a host plant for these organisms in winter [13,14,15]. Costello [16] reported the results of research on broccoli grown with no cover crop or with living mulches (white clover, strawberry clover, and mixture of birdsfoot trefoil and red clover) and showed that the aphid infestation was less in living mulches than under clean cultivation. Moreover, when fertilized with compost, yield was higher with living mulches than in clean cultivation. Pieper et al. [17] reported that perennial white clover provided yields of carrot, melon, and cucumber, similar to the control, but reduced yields of tomato, cabbage, and lettuce; however, microbial respiration was higher while loss of soil organic matter was lower. In organic spinach production, a linear relationship between biomass of weeds and subterranean clover was found by Bàrberi et al. [18].

In these crop systems it is important to prevent competition between crop and living mulches for light, moisture, and nutrients, which in most cases cause yield reduction of cash crops [8,19]. For example, Hiltbrunner et al. [20] suggested that legumes producing more dry matter (e.g., *Trifolium repens*) control weeds better than species producing less dry matter (e.g., *T. subterraneum*). However, also the competition between crop and living mulch, estimated by winter wheat biomass at anthesis, increases with increasing cover crop dry matter. In addition, Pfeiffer et al. [21] reported that living mulch may contribute to weed suppression in vegetable production. Moreover, lower yields were recorded in the living mulch treatments, most likely due to competition among vegetables, living mulches, and weeds, especially in case of high pre-existing weed seedbank and drought conditions. 

Organic farming techniques that preserve soil health can add further value to traditional species and landraces of vegetables, linking the objective of preserve agro-biodiversity with environment protection. In addition, in Italy, the organic farms of Puglia (the most important region for the production of organic vegetables) are usually of small dimension and often without livestock production. Consequently, under these conditions, it can be very difficult to preserve and/or improve soil fertility. Thus, the use of cover crops can make a valid contribution to making organic farming production possible and to preserving soil health. 

Broccoli raab (*Brassica rapa* L. subsp. *sylvestris* L. Janch. var. *esculenta* Hort) is an ancient vegetable of Mediterranean origin, linked to the culinary traditions of a large part of Central-Southern Italy. This vegetable is mainly cultivated in Southern Italy but in the last decades has attracted the attention of an increasing number of consumers in Europe, the United States, Canada, Argentina, and Australia. The popularity of this typical vegetable of Puglia is probably due both to its aromatic taste and content of glucosinolates, well-known as important health compounds [22]. As a consequence of its popularity in Puglia and thanks to the long work of selection carried out by farmers, a consistent number of broccoli raab landraces are disseminated throughout the region. These are characterized by large variability, especially in relation to the cycle length (mostly intended as the time period between planting and the appearance of the main inflorescence) [22,23]. Populations can be divided into early, mid, and late varieties. They take their name from the area of cultivation or the length of the crop cycle, from the most likely time of harvest, from the size of the inflorescence, or from two or more of the above features [22,24].

Although the Puglia is the most important Italian region for the cultivation of broccoli raab, to the best of our knowledge there is a lack of information in literature with regard to this vegetable grown under organic production systems. Therefore, starting from all the above remarks, the aims of this research were: (i) to investigate the influence of living mulch on both weed infestation and broccoli raab production; (ii) to evaluate the effects of organic fertilization by applying different rates of nitrogen and phosphorous on yield and quality of broccoli raab. The general goal was to assess the obtaining of a sustainable and high-quality production of this *Brassicaceae* species.

## 2. Materials and Methods 

### 2.1. Cropping Details

Broccoli raab (cv “Grossa fasanese”) was cultivated in a field located in Noicattaro (Puglia, Southern Italy). Transplanting was carried out on October 2017, while harvesting was carried out on February 2018. Characteristics of the soil were: (i) clay-loam texture (clay 34%, sand 38%, and silt 28%); (ii) sub-alkaline reaction (pH 7.5); (iii) total N 1.98‰; (iv) assimilable P_2_O_5_ 18.5 mg kg^−1^; (v) exchangeable K_2_O 825 mg kg^−1^; (vi) assimilable Fe 8.43 mg kg^−1^; (vii) organic matter content 1.5%; (viii) CEC 26.9 meq 100 g^−1^.

Four soil management systems (SMSs) were evaluated as follows: living mulch with *Trifolium subterraneum* cv Clare (LMS); *Trifolium repens* cv Huia (LMR); undisturbed weedy (UN); conventional tillage (CT). For each of the SMSs, four rates of nitrogen and phosphorous (NP) were supplied (kg ha^−1^): 0 N + 0 P_2_O_5_ (NP0); 42 N + 78 P_2_O_5_ (NP1); 84 N + 156 P_2_O_5_ (NP2); 126 N + 234 P_2_O_5_ (NP3). Organic fertilizer, composed of meat and bone meal (7% N and 13% P_2_O_5_ - Regenor NP©), was used.

The experimental treatments were arranged according to a split-plot design with three replications, where SMSs were set in the main plots (12.0 × 8.0 m) and NP in the sub-plots (6.0 × 4.0 m).

The experimental field, previously cultivated with table grape, was not cultivated for five years. Before the sowing of living mulch, in all plots, soil was ploughed at 30 cm and subsequently tilled with a rotary harrow. The two species of clover were broadcast seeded by hand on 16 October 2017, covering seeds by a second rotary harrowing. Seed rates were 30 kg ha^−1^ for *T. subterraneum* and 10 kg ha^−1^ for *T. repens*. Fertilization and transplanting (distance of 0.6 m between the rows and 0.5 m on the row) of broccoli raab were done two days after the sowing of clovers. Ten days after sowing the emergence of more than 60% of clovers was observed. In CT plots, weeds were mechanically controlled (rotary harrowing) 30, 50, and 70 days after transplanting, whereas no weed control was performed in the other plots. 

### 2.2. Evaluation of Weed Infestation

In December, weed infestation in each plot was evaluated using the phytosociological method of Braun-Blanquet [25]. The cover-abundance values of each species were recorded according to the original Braun-Blanquet scale, but they were converted into the ordinal scale proposed by Van der Maarel [26]. Only the total cover, obtained as the sum of the cover of all species, will be shown.

### 2.3. Yield and Physical Analysis

From January to February, broccoli raab inflorescences (primary and secondary) of 30 plants in each plot were harvested, as they reached edible and marketability characteristics. Weight, diameter (both at the middle and at the base of cut), and colour were measured for the main inflorescences. The total weight of inflorescences (primary and secondary) and leaves around their base (normally edible) was assumed as marketable yield.

Colour parameters were measured with the colorimeter (CR-400, Konica Minolta, Osaka, Japan) equipped with illuminant D65, in reflectance mode and with CIE L (lightness), a* (redness) and b* (yellowness) colour scale. L, the lightness value, ranging from black = 0 to white = 100; a*, ‘red/green chromaticity’, red-violet colour if positive, green-blue colour if negative; b*, ‘yellow/blue chromaticity’, yellow colour if positive, blue colour if negative. Before the readings were performed, the colorimeter was calibrated with a standard reference with L, a*, and b* values of 97.38, 0.08, and 1.89, respectively. Hue angle (h° = arctg b*/a*) and saturation (Chroma = [a*^2^ + b*^2^]^1/2^) were then calculated from primary L, a*, and b* readings. Measures were performed on the inner part of the half-cut inflorescences, recording the mean value of the 30 data collected.

Dry weight was measured on ten primary inflorescences, randomly harvested in each plot, and dried in an oven at 40 °C. A sample was also collected in each plot and freeze-dried for the determination of anion and mineral components.

### 2.4. Sufficient Index Calculation

A Minolta SPAD-502 m (Minolta, Ramsey, N.J.) was used to take chlorophyll readings. Measurements were performed after one and two months on 20 external leaves randomly chosen in each plot. A Sufficiency Index (SI) was calculated as reported in Petersen et al. [27] except that the highest average treatment was used as the maximum. In our study, the SI was calculated as follow:

SI (%) = (SPAD–based plot readings/Highest-fertilized plot SPAD readings) × 100

The Sufficient Index is a useful tool to estimate N availability in plants, considering that a SPAD reading is non-destructive and allows repeated estimations during the growing season, highlighting potential N deficiency early.

### 2.5. Chemical Analysis

For anion content determination, the ion exchange chromatography (Dionex DX120, Dionex Corporation, Sunnyvale, CA, USA) with a conductivity detector was performed as reported by Gonnella et al. [28]. Nitrate, Cl, SO_4_, and H_2_PO_4_ and oxalate contents were determined in 0.5 g of dried sample using an IonPac AG14 precolumn and an IonPac AS14 separation column (Dionex Corporation).

Determination of Mg, Fe, Na, K, and Ca content was performed according to the official methods of analysis [29]. Briefly, about 0.5 g of dried sample, in triplicates, was accurately weighed and transferred into a Kjeldahl flask, digested with 5 mL of HNO_3_ and 1 mL of H_2_O_2_, then filled up to the volume of 50 mL with deionized water. All measurements were performed using an Agilent MP-AES 4200 instrument, with nitrogen supplied from an Agilent 4107 Nitrogen Generator, with the standard sample introduction system consisting of the OneNeb nebulizer, double pass cyclonic spray chamber, and easy fit torch. An External Gas Control Module (EGCM) accessory and an SPS four autosampler were also used. The MP-AES was controlled using the MP Expert software, which recommends wavelengths for the selected elements and automatically sets the nebulizer flow rate and EGCM settings. Auto background correction was used to resolve the element emission line from the organic matrix. The Agilent SPS-3 auto-sampler was used to deliver samples to the instrument allowing unattended operation. Selection of optimal lines depended on wavelengths that were free from spectral interference and matched the appropriate sensitivity. Spectral and background interferences could be simultaneously and accurately corrected for using the MP software. Standard reference solutions used in the external calibration method were prepared at concentrations of 1 and 10 mg/L, from a 100 mg/L multi-element calibration standard. At the last harvest, the total above-ground biomass (four plants from each plots) was cut and weighted. 

### 2.6. Statistical Analysis

Statistical analysis was carried out using the GLM (General Linear Model) procedure (SAS Software, Cary, NC, USA) using a split-plot experimental design in a two-way analysis of variance (ANOVA). Duncan’s test was used to establish differences between means.

## 3. Results

For all the measured parameters, ANOVA revealed that the interaction between soil management and fertilization rates was not significant (Table 1, Table 2 and Table 3). Therefore, only significant data of principal factor will be reported in other tables and figures.

The most abundant weeds recorded in the plots were *Vicia villosa* Roth, *Medicago polymorpha* L., *Cirsium arvense* (L.) Scop., *Lathyrus* sp., *Veronica hederifolia* L., *Geranium molle* L., *Lamium amplexicaule* L., and *Chenopodium album* L. Plots with living mulch and CT showed the lowest level of infestation (Table 4).

Both 30 and 60 days after transplanting, unfertilized (NP0) broccoli raab plants showed the lowest Sufficient Index (SI); moreover, with increasing organic fertilization, the Sufficient Index linearly increased. Above-ground biomass and marketable yield linearly increased with the increasing of fertilization levels (Table 5).

With increasing organic fertilization rates, also the weight of primary inflorescences linearly increased (Table 6). NP3 and NP2 showed the highest value of diameter (both base of the cut and equatorial) and the lowest value of dry matter of primary inflorescences (Table 6).

Regarding anions, NO_3_ content was about 5-fold higher in NP1 than in NP0, while no significant differences were found between NP2 and NP3 in comparison with all other fertilization levels (Table 7). NP0 and NP1 showed a SO_4_ content 34% higher than NP2 and NP3. Cl content was highest in NP0, while the content of oxalate was, on average, 59.9 mg kg^−1^ without any difference among fertilization treatments (Table 7).

The content of Mg in NP3 was 48% higher than NP0 and NP2, while no differences were found between NP1 and all other treatments (Table 8). Similarly, the content of iron in NP3 was 3.5-fold higher than NP0, while no differences were found between NP1 and NP2 in comparison with all other treatments. NP0 and NP1 samples showed a content of Na, K, and Ca higher respectively of 30, 22 and 21% than NP2 and NP3 samples (Table 8).

As regard the colour parameters, L and a* values were 43.5 and −13.8, respectively, without any differences among fertilization levels (Table 9). Both b* and C values were higher in NP0 and NP1 than NP3, while no differences were found between NP2 and the other treatments. On the other hand, the h° value in NP0 and NP1 was lower by about one unit than NP2 and NP3 (Table 9).

## 4. Discussion

This study investigated the performance of living mulch, an agronomic technique considered of great applicative interest in the organic production systems of vegetable crops. Its effects were also investigated in relation to nitrogen and phosphorous organic fertilization.

Both the clover species used in the trial proved to be well adapted to the climate and soil characteristics of the experimental field, forming high canopy that suppressed the growing of weeds, to the point that their abundance was similar to that found in the tilled plots. These results confirm the adaptability of *T. subterraneum* and *T. repens* also in vegetable crops, as reported for orchards in the same Mediterranean environment by Corleto and Cazzato [30] and Torres et al. [31].

Competition is a critical issue in these cultivation systems, because living mulch can compete with vegetables for light, moisture, and nutrients, causing yield reduction [8]. Considering only the effect of living mulch, in our experiment, the qualitative-quantitative parameters measured on the crop did not reveal noteworthy negative effects regarding competition with broccoli raab, although weeds were controlled. Furthermore, the SPAD indices, in the early crop stages, were not affected by clovers. These findings are consistent with those showed in the meta-analysis performed by Verret et al. [32] about legume companion plants (including *T. repens* and *T. subterraneum*), because they did not find significant effects on the yield of several cash crops (maize, wheat, barley, oat, etc.), although weed infestation decreased. Canali et al. [33] report in cauliflower that living mulch does not reduce yield and yield quality and creates an unfavorable environment for weeds, avoiding crop suppression.

Den Hollander et al. [19] report results in a study about several clovers used as cover crop for weed suppression in leek. It has been concluded that even subterranean clover and white clover, although the least competitive species among those investigated, caused reductions in individual plant weight of leek. The reason of the considerable competitive effect was that in the experiment the leek plants were completely entangled within the clover canopy. It is important to note that effects on transplanted leek were strongly correlated with canopy height, indicating that yield reduction was mainly caused by competition for light. In our experiment, we sowed clovers simultaneously to the transplanting of crop; therefore, they were unable to cover plants and compete for light. On the contrary, in a similar experiment that we performed on tomato transplanted in May, while the clovers were actively growing, the crop was completely entangled, and it suffered tough competition from *T. subterraneum* and *T. repens* (unpublished data). In addition, Adamczewska-Sowińska et al. [11] found a significant drop in the yield of tomato for the earliest sowing date of clovers, while later sowing dates of white clover gave yields at the same or even higher level than in the control.

Organic fertilization showed prominent effects on most of the characteristics evaluated. It showed its effects from the early stages, when the SI of broccoli raab plants was higher in fertilized plots. Peterson et al. [27] reported that a SI lower than 95% indicates an N deficiency that may lead to a yield reduction. Effectively, NP1 treatment (SI = 93.5, 60 days after transplanting—Table 5) showed that marketable yield and weight of primary inflorescences of broccoli raab were not different from the NP0 treatment (SI = 91.0, 60 days after transplanting—Table 5), while with increasing fertilization rates the yield increased linearly. Literature lacks information regarding the effects of N fertilization on crop performance of broccoli raab. On the other hand, in a study aimed to evaluate the influence of nitrogen fertilization on broccoli (*Brassica oleracea* var. *italica*) yield, Zebarth et al. [34] found that the marketable yield increased curvilinear by increasing the rate of N fertilization. Our results confirm the great influence of N fertilization on yield of the *Brassicaceae* vegetables, highlighting the importance of combining living mulch and fertilization in a context of organic farming techniques. 

Apart from yield, it is interesting to note that by increasing fertilization rates of N and P, we observed an increase of some elements such as Mg and Fe as well as a decrease of other ones such as Na, K, and Ca. Furthermore, dry matter decreased when the two highest rates of organic fertilization were used. 

As regards the nitrate content, we observed differences only between the fertilized plots and unfertilized ones. To this end, it must be considered that for all treatments the nitrate content in edible portions of broccoli raab was very low. Effectively, Santamaria [35] defined the nitrate content in vegetables as “very low” when the amount is lower than 200 mg kg^−1^ FW, while he classified broccoli raab as a vegetable with a “middle content” of nitrate (500‒1000 mg kg^−1^ FW). Nitrate content is an important quality trait of vegetables, considering that a low amount is preferred for preventing potential negative effects on human health [35]. There are several factors affecting nitrate uptake and accumulation in vegetable tissues such as genetic, environmental, and agricultural ones. Our results suggest that by combining living mulch and organic fertilization strategies it is possible to obtain broccoli raab with a very low content on nitrate. On the other hand, it should be considered that nitrate content in vegetables changes in the different plant parts in a decreasing manner as follows: petiole > leaf > stem > root > inflorescence > tuber > bulb > fruit > seed [35]. In this context, it is important to specify that in our study we considered only broccoli raab inflorescences an edible portion by reducing the presence of leaves and stems. Regarding rocket (*Diplotaxis tenuifolia* L.), Santamaria et al. [36] identified the harvesting of only leaf lamina without petiole as a way of reducing the nitrate content in the edible portion, considering that the nitrate content in rocket petioles can be more than two-fold higher in comparison with leaf lamina [36]. Therefore, our study highlighted the possibility of harvesting only the inflorescences of broccoli raab as a way of reducing the nitrate content in the edible portion.

As regards the colour of broccoli raab, it should be considered that this trait represents the first quality parameter evaluated by consumers, and it is critical to product acceptance. In more detail, hue angle, considered the qualitative attribute of colour, is the attribute that has traditionally defined the colours as reddish, greenish, etc., and it is used to define the difference of a certain colour with reference to grey colour of the same lightness. A hue angle of 0 or 360° represents red hue, while angles of 90°, 180°, and 270° represent yellow, green, and blue hues, respectively [37]. Thus, a higher hue angle in NP2 and NP3 samples indicates a greener colour with respect to both lower fertilized samples and unfertilized ones. This result is in agreement with the highest SPAD values of NP2 and NP3 samples, indicating a higher chlorophyll content in these samples than in the others. Thus, the results of our study suggest that different organic fertilization strategies can significantly affect an important quality trait in broccoli raab, such as colour. 

## 5. Conclusions

Living mulch gave positive effects on weed management, without affecting yield and quality of broccoli raab: the lowest level of infestation was obtained with *Trifolium subterraneum* (better than that obtained with conventional tillage). Weight and size of broccoli raab inflorescences and marketable yield linearly increased with the increasing of organic fertilization levels. As regards quality, by using higher organic fertilization rates we found a greener colour, highlighting the importance of the fertilization on an important quality trait for consumers, such as colour. From a nutritional point of view, nitrate (potentially negative for human health) content was very low in broccoli raab inflorescences for all organic fertilization treatments. In conclusion, the results of this study suggest the positive effects of living mulch and organic fertilization to obtain a sustainable and high-quality production of broccoli raab. 

## Figures and Tables

**Table 1:** Significance of soil management system, organic fertilization rates, and their interaction on weed infestation, Sufficient Index, total above-ground biomass, marketable yield, weight, size, and dry matter of primary inflorescences of broccoli raab.

Treatment.	Total Infestation	Sufficient Index		Total above-Ground Biomass	Marketable Yield	Primary Inflorescences			
						Weight	Diameter		Dry Matter
		30 Days after Transplanting	60 Days after Transplanting				Base of the Cut	Equatorial	
**Soil Management**	*	**ns**	**ns**	**ns**	ns	ns	ns	ns	ns
Fertilization rates	ns	***	***	***	***	***	***	***	***
Interaction	ns	ns	ns	ns	ns	ns	ns	ns	ns

Significance: ns, not significant; * *P* < 0.05; ** *P* < 0.01; *** *P* < 0.001.

**Table 2:** Significance of soil management system, organic fertilization rates, and their interaction on anion content and colour parameters of primary inflorescences of broccoli raab.

Treatment	Anion Content				Colour Parameters				
	NO_3_	SO_4_	Cl	Oxalate	L	a*	b*	h°	C
Soil Management	ns	ns	ns	ns	ns	ns	ns	ns	ns
Fertilization rates	*	**	***	ns	ns	ns	**	**	**
Interaction	ns	ns	ns	ns	ns	ns	ns	ns	ns

Significance: ns, not significant; * *P* < 0.05; ** *P* < 0.01; *** *P* < 0.001.

**Table 3:** Significance of soil management system, organic fertilization rates, and their interaction on Mg, Fe, Na, K, and Ca content of primary inflorescences of broccoli raab.

Treatment	Elements				
	Mg	Fe	Na	K	Ca
Soil Management	ns	ns	ns	ns	ns
Fertilization rates	*	ns	***	***	**
Interaction	ns	ns	ns	ns	ns

Significance: ns, not significant; * *P* < 0.05; ** *P* < 0.01; *** *P* < 0.001.

**Table 4:** Soil management system and total infestation of broccoli raab crop.

Soil Management Systems ^(1)^	Total Weed Coverb^(2)^ (%)
CT	4.9 b
UN	17.3 a
LMS	0.9 b
LMR	6.3 b

(1) Living mulch with *Trifolium subterraneum* (LMS); living mulch with *Trifolium repens* (LMR); undisturbed weedy (UN); conventional tillage (CT). (2) Data followed by different letters are significantly different at *P* < 0.05.

**Table 5:** Effects of organic fertilization rates on Sufficient Index, total above-ground biomass, and marketable yield of broccoli raab ^(1).^

8	Sufficient Index (%)		Total Above-Ground Biomass (t ha^−1^)	Marketable Yield (t ha^−1^)
	30 Days after Transplanting	60 Days after Transplanting		
NP0	88.2 c	91.0 b	7.6 d	3.0 c
NP1	92.5 b	93.5 b	12.3 c	4.0 c
NP2	93.9 b	98.4 a	18.5 b	6.7 b
NP3	99.4 a	100.0 a	23.9 a	8.1 a

(1) 0 N + 0 P_2_O_5_ kg ha^−1^ (NP0); 42 N + 78 P_2_O_5_ kg ha^−1^ (NP1); 84 N + 156 P_2_O_5_ kg ha^−1^ (NP2); 126 N + 234 P_2_O_5_ kg ha^−1^ (NP3). Data followed by different letters are significantly different at *P* < 0.05.

**Table 6:** Effects of organic fertilization rates on weight, size (diameters at the base of the cut and equatorial), and dry matter of primary inflorescences ^(1)^.

Fertilization Rates	Weight (g)	Diameter (mm)		Dry Matter (g 100 g^−1^ Fresh Weight)
		Base of the Cut	Equatorial	
NP0	59.1 d	24.5 c	38.8 c	11.0 a
NP1	78.7 c	27.1 b	43.1 b	10.1 a
NP2	122.1 b	31.5 a	50.3 a	8.5 b
NP3	149.0 a	32.7 a	53.1 a	8.3 b

(1) 0 N + 0 P_2_O_5_ kg ha^−1^ (NP0); 42 N + 78 P_2_O_5_ kg ha^−1^ (NP1); 84 N + 156 P_2_O_5_ kg ha^−1^ (NP2); 126 N + 234 P_2_O_5_ kg ha^−1^ (NP3). Data followed by different letters are significantly different at *P* < 0.05.

**Table 7:** Effects of organic fertilization rates on the content of anion components (mg kg^−1^ of fresh matter) of the inflorescences ^(1)^.

Fertilization Rates	NO_3_	SO_4_	Cl	Oxalate
NP0	10.6 b	574 a	627 a	57.3
NP1	63.6 a	498 a	428 b	60.4
NP2	26.3 ab	402 b	390 b	64.4
NP3	34.8 ab	397 b	434 b	57.6

(1) 0 N + 0 P_2_O_5_ kg ha^−1^ (NP0); 42 N + 78 P_2_O_5_ kg ha^−1^ (NP1); 84 N + 156 P_2_O_5_ kg ha^−1^ (NP2); 126 N + 234 P_2_O_5_ kg ha^−1^ (NP3). Data followed by different letters are significantly different at *P*< 0.05.

**Table 8:** Effects of organic fertilization rates on the content of Mg, Fe, Na, K, and Ca (mg kg ^−1^ of fresh matter) of the inflorescences ^(1)^.

Fertilization Rates	Mg	Fe	Na	K	Ca
NP0	86.4 b	1.9 b	2771 a	2933 a	352 a
NP1	104.3 ab	2.5 ab	2594 a	2769 a	349 a
NP2	81.8 b	7.0 ab	2157 b	2359 b	294 b
NP3	124.5 a	8.7 a	2068 b	2295 b	288 b

(1) 0 N + 0 P_2_O_5_ kg ha^−1^ (NP0); 42 N + 78 P_2_O_5_ kg ha^−1^ (NP1); 84 N + 156 P_2_O_5_ kg ha^−1^ (NP2); 126 N + 234 P_2_O_5_ kg ha^−1^ (NP3). Data followed by different letters are significantly different at *P* < 0.05.

**Table 9:** Effects of fertilization on colour parameters of primary inflorescences ^(1)^.

Fertilization Rates	Colour Parameters				
	L	a*	b*	h°	C
NP0	43.1	−14.0	20.7 a	124.0 b	25.0 a
NP1	44.1	−14.1	20.6 a	124.4 b	25.0 a
NP2	43.6	−13.9	19.6 ab	125.3 a	24.0 ab
NP3	43.3	−13.4	18.8 b	125.5 a	23.1 b

(1) 0 N + 0 P_2_O_5_ kg ha^−1^ (NP0); 42 N + 78 P_2_O_5_ kg ha^−1^ (NP1); 84 N + 156 P_2_O_5_ kg ha^−1^ (NP2); 126 N + 234 P_2_O_5_ kg ha^−1^ (NP3). Data followed by different letters are significantly different at *P* < 0.05.
